# Novel method for simultaneously detecting HPA and HLA antibodies using Luminex microbeads

**DOI:** 10.1186/s12967-019-2002-4

**Published:** 2019-08-05

**Authors:** Sudan Tao, Shu Chen, Xiaozhen Hong, Ji He, Faming Zhu

**Affiliations:** 1 Blood Center of Zhejiang Province, Jianye Road 789, Hangzhou, 310052 Zhejiang China; 2 Key Laboratory of Blood Safety Research, Zhejiang Province, Jianye Road 789, Hangzhou, 310052 Zhejiang China

**Keywords:** Luminex assay, HPA antibodies, HLA antibodies, MAIPA

## Abstract

**Background:**

Alloantibodies against human platelet antigens (HPAs) and human leukocyte antigen (HLA) are implicated in several immune-mediated platelet disorders. Detection of these antibodies is crucial in the diagnosis and management of these disorders. The aim of this study was to establish a novel method to simultaneously detect HPA-1, HPA-2, HPA-3, HPA-5 and HLA antibodies with Luminex microbeads technology.

**Methods:**

Monoclonal antibodies specific for platelet glycoproteins and HLA class I molecules were separately coupled to the Luminex microbeads. We validated specificity of the Luminex platform using the following antibodies: anti-HPA-1a, anti-HPA-2b, anti-HPA-3a, anti-HPA-5a, and anti-HLA positive samples. Sensitivity was evaluated by a serial dilution (from neat to 1/1024) using the following antibodies: anti-HPA-1a, anti-HPA-3a standard sera, and anti-HPA-5a positive serum. Serum samples were collected from 36 neonatal alloimmune thrombocytopenia (NAIT) patients suspected of having HPA or HLA antibodies and 8 samples from ISBT platelet workshop were tested using the Luminex assay.

**Results:**

The Luminex assay detected all antibodies tested from the known samples. The sensitivities of the Luminex assay detecting anti-HPA-1a, anti-HPA-3a, and anti-HPA-5a were 1:512, 1:64, and 1:128, respectively. The sensitivity of Luminex assay was higher than monoclonal antibody immobilization of platelet antigen method (MAIPA). No cross-reactivity was observed in the samples containing multi-platelet antibodies or mixture antibodies against HPA and HLA. The results of 44 samples with platelet disorders were consistent with those of the same samples processed with the MAIPA assay.

**Conclusion:**

Luminex microbeads coupled with monoclonal antibodies could be successfully used to detect HPA and HLA antibodies simultaneously, especially with high sensitivity in detecting HPA antibodies.

## Background

Alloantibodies against human leukocyte antigen (HLA)-I class and human platelet antigen (HPA) are involved in several immune-mediated platelet disorders including neonatal alloimmune thrombocytopenia (NAIT), post-transfusion purpura (PTP), and platelet transfusion refractoriness (PTR) [1–4]. Most of PTR is a result of HLA alloreactivity. In addition, HLA antibodies are regarded as the main cause of transfusion-related acute lung injury [5] and associated with rejection and graft dysfunction after solid-organ transplantation and hematopoietic stem cell transplantation. HPA antibodies are also associated with platelet immune disease. HPA systems (http://www.ebi.ac.uk/ipd/hpa/) mainly distribute on the corresponding platelet membrane glycoproteins (GP), including GPIIb/IIIa, GPIb/V/IX, GPIa/IIa, and CD109. Of the 29 HPA systems, only HPA-1-6, HPA-15, and HPA-21w systems are polymorphic in the Chinese Han population, which may correlate with alloimmune platelet disorders. Detection of antibodies against HLA and HPA is essential in the diagnosis and management of these disorders.

There are several methods to detect HPA antibodies, including solid phase red cell adherence (SPRCA), monoclonal antibody specific immobilization of platelet antigen (MAIPA) technology, immuno-complex capture fluorescence analysis (ICFA), gel antigen-specific assay (GASA), and HP cell-based monoclonal antibody-independent Immobilization of Platelet Antigen [6–8]. However, the inter-laboratory quality assessment (EQA) project shows that there are discrepancies in the results using different experimental methods. Therefore, it is necessary to update these methods to effectively identify HPA antibody, which is important for the clinical diagnosis of HPA antibody and the improvement of platelet transfusion efficiency.

Assays using the Luminex technology have been developed to detect HPA antibodies and identify antibody types. Detection of the HPA-1 system has been developed by coupling soluble recombinant platelet glycoprotein to Luminex beads [9]. Some HPA antibodies have been detected by coupling a purified platelet membrane glycoprotein to Luminex beads [10]. These studies showed that Luminex technology is more sensitive, rapid and higher throughput than other methods. While HLA antibody detection methods such as Luminex-PRA are well described, simultaneous detection of HPA and HLA has still not been implemented. In this study, we established a novel method using the Luminex platform to simultaneously detect HLA and HPA antibodies (HPA1-3, HPA-5, and HPA-15). We coated Luminex microbeads with monoclonal antibodies specific to platelet glycoproteins and HLA class I molecules. The Luminex assay was compared with the MAIPA and PRA methods to evaluate its specificity and sensitivity.

## Methods

### Monoclonal antibodies and reagent

Monoclonal antibodies: anti-GPIb/IX (clone: AK2, Santa Cruz, Texas, USA), anti-GPIIb/IIIa (clone: P2, Acris, San Diego, USA), anti-GPIa/IIa (clone: Gi9, Beckman Coulter, Atlanta, USA), anti-CD109 (clone: W7C5, MBL, Woburn, USA), anti-HLA (Clone: W6/32, Abcom, Cambridge, the United Kingdom), biotin conjugated mouse anti-human IgG (Clone: M148, Acris, San Diego, USA), biotin conjugated goat anti-mouse monoclonal IgG (Abcom, Rockville, USA). LABScreen® PRA Class I kits (One Lambda, USA) were used to detect HLA antibodies.

### Serum samples

Forty-four serum samples including 8 samples from ISBT platelet workshop and 36 samples previously collected from patients suspected of having HPA or HLA antibodies were tested in this study. Three standard sera containing anti-HPA-1a (National Institute of Biological Standards and Control code: 03/152), anti-HPA-3a (NIBSC code: 03/190), and anti-HPA-5b (NIBSC code: 99/666), respectively, were used as positive controls to validate the specificity and sensitivity of HPA antibody detection. Another 163 serum samples collected from uremia patients were tested by Luminex-PRA to evaluate the specificity of the Luminex assays detecting HLA antibodies. Serum samples from 8 AB phenotype healthy blood donors were used as negative controls.

### Luminex assay

**Fig. 1:**
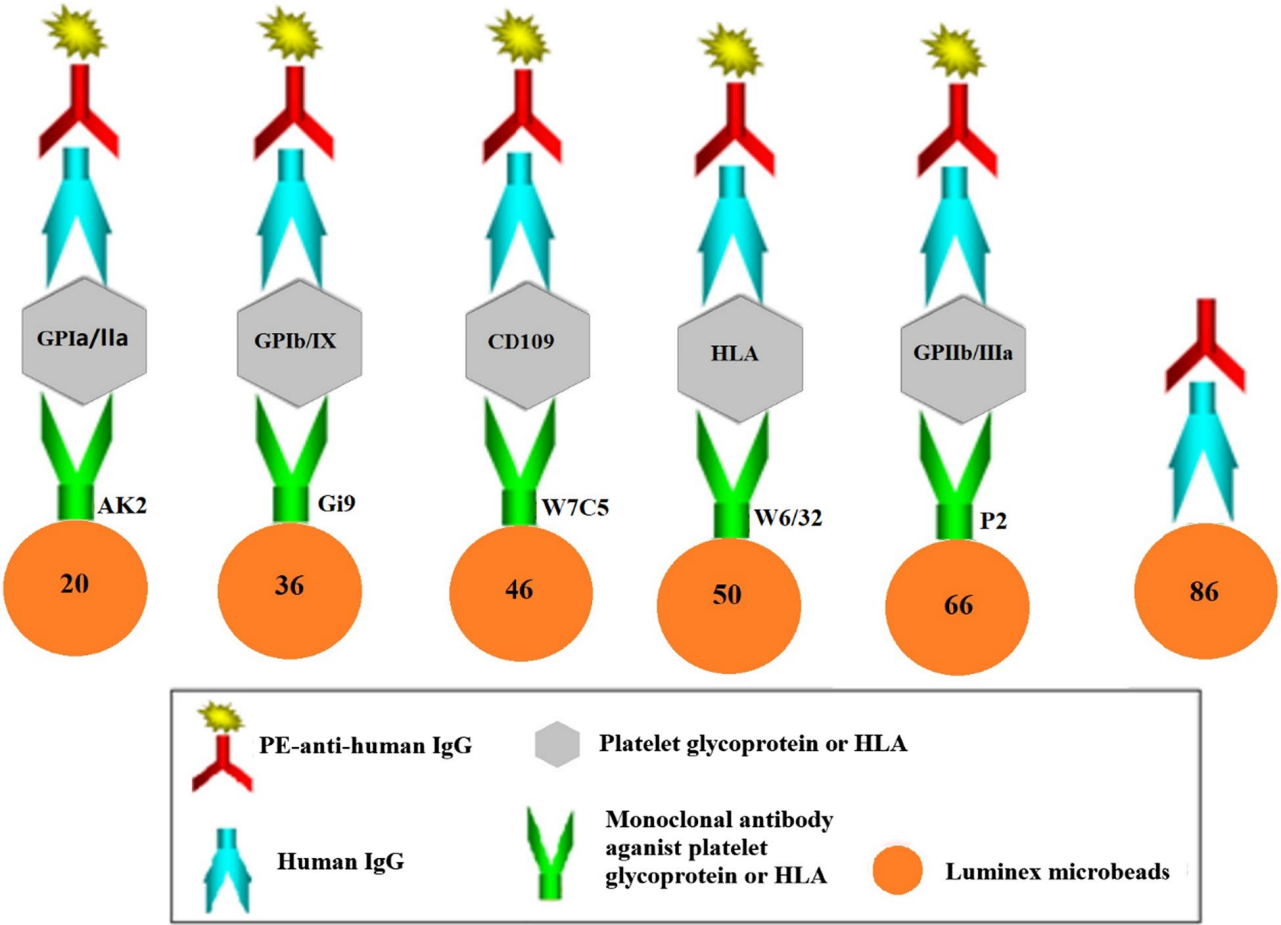
An overview of the Luminex assay

Monoclonal antibodies P2, Gi9, AK2, W7C5 and W6/32 (5 μg) specific for GPIIb/IIIa, GPIb/IX, GPIa/IIa, CD109 and HLA were separately added to 5.0 × 10^6^ Luminex xMAP beads (LC10066-01, LC10020-01, LC10036-01, LC10046-01, and LC10050-01), and quantified to 500 μL buffer solution (50 mmol/L 2-morpholino ethanesulfonic acid), then incubated for 2 h in room temperature. The mixtures were then centrifuged and washed 2 times with PBS-TBN buffer (PBS, 0.1% BSA, 0.02% Tween-20, 0.05% azide, pH 7.4). The coupled microbeads were vortexed and sonicated with 1 mL PBS and counted. The coupled microsphere stocks were diluted to a final concentration of 1 × 10^3^ beads/μL in assay buffer. The overview of the Luminex assay has been showed in Fig. 1. Human IgG was coupled to beads LC10086-01 as a positive control.

Fresh apheresis platelets were collected from three voluntary blood donors (P2, P3 and P4) and genotyped for HPA as previously described [11]. The platelets were centrifuged at 1400*g* for 5 min at 4 °C, washed 2 times with TBS-EDTA buffer, and resuspended with TBS-EDTA buffer. We reacted 1 × 10^7^ platelets with 25 μL serum samples at 37 °C for 30 min. Following incubation, the samples were centrifuged at 1400*g* for 3 min and washed 2 times with TBS-EDTA. We then added 50 μL lysate buffer (20 mmol/L Tris contain 0.1% Triton X-100, 3 mmol/L EDTA, 150 mmol/L NaCl) and lysed at 4 °C for 30 min. After centrifugation at 1400*g* for 45 min, the supernatant lysates were collected. Each antibody-platelet complex lysate (30 μL) was reacted with 10 μL microbead mixtures at 37 °C, 110 rpm vibration for 30 min. After washing (3 times with PBS-TBN), 100 μL of phycoerythrin-conjugated goat anti-human IgG was added and reacted at 37 °C, 110 rpm vibration for 30 min. Following another wash (3 times with PBS-TBN), 100 μL PBS-TBN buffer was added and subjected to flow cytometric analysis on a Luminex 100 instrument. All the serum samples were also tested by MAIPA. The anti-HPA-1a, anti-HPA-3a standard sera, and anti-HPA-5a positive samples were serially diluted (from 1:1 to 1:1024) for the sensitivity assay.

### Confirmation of bead coupling

Monoclonal antibodies P2, Gi9, AK2, W7C5 and W6/32 specific for GPIIb/IIIa, GPIa/IIa, GPIb/IX, CD109 and HLA were separately coupled to the carboxyl group of microbeads through –NH2 peptides. The coupled beads were validated by goat anti-mouse monoclonal IgG. After staining with biotin-conjugated goat anti-mouse monoclonal IgG, PE-conjugated streptavidin was added to the mixture to measure MFI (Luminex 100).

### Cut-off index

The mean fluorescence value of each well was detected on a Luminex 100 instrument. The cut-off value is defined as the mean value of the negative control + 3SD value, and the ≥ cut-off value is considered as positive [12].

## Results

### Monoclonal antibody-coupled beads confirmation

The coupled beads were validated by goat anti-mouse monoclonal IgG. The MFI values of coupled bead reacted with goat anti-mouse are 2661 ± 327 for anti-GPIa, 7871 ± 585 for GPIba, 4573 ± 399 for GPIIb/IIIa, 4471 ± 283 for CD109, 3741 ± 89 for HLA, 4831 ± 27 for human IgG, and 20 ± 1 to 40 ± 7 for empty beads (negative control). The coupled beads exhibited extremely higher MFI values than that of the empty beads, which indicated all the monoclonal antibodies had been successfully coupled to the microbeads.

### Specificity and sensitivity of Luminex assay detecting HPA antibodies

**Fig. 2:**
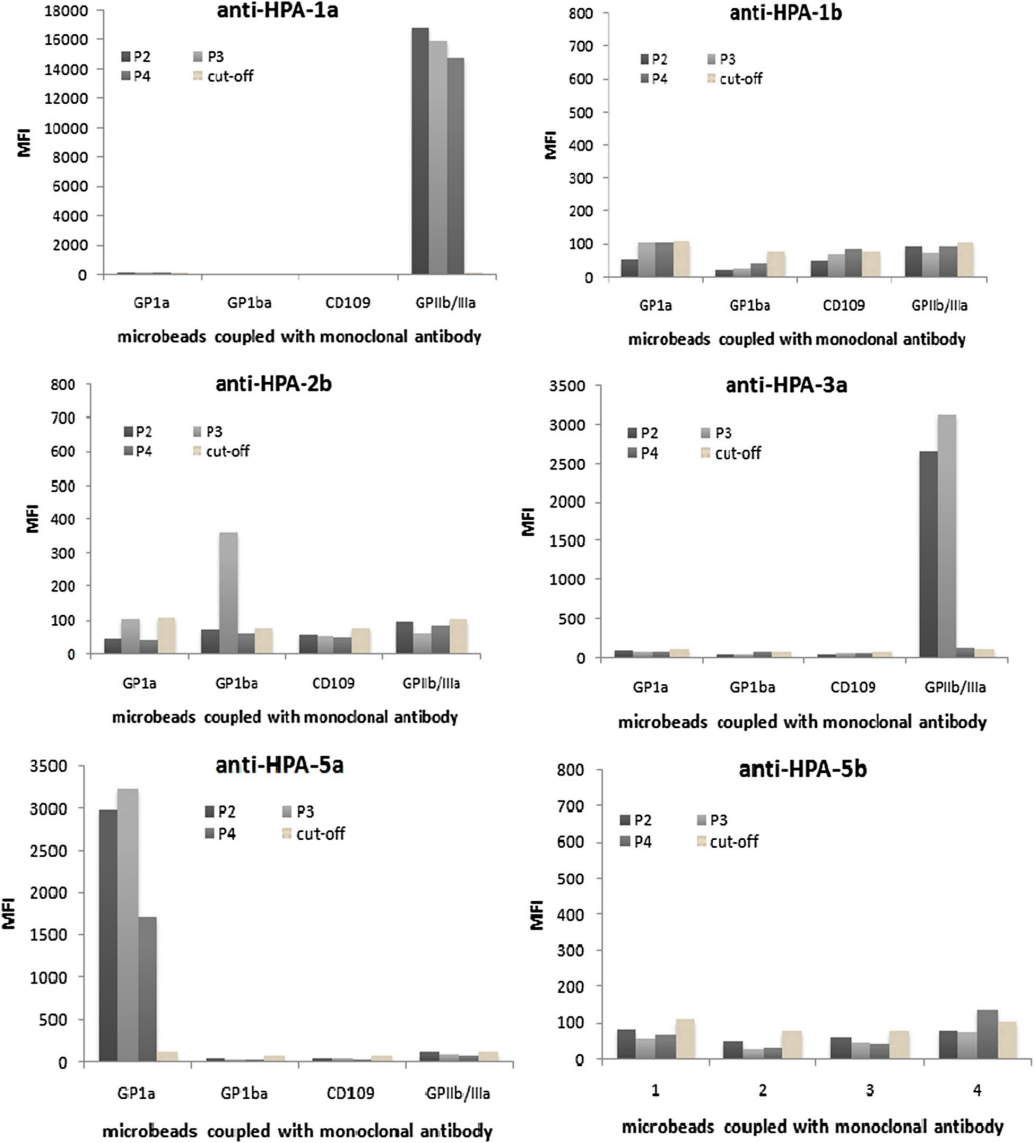
Detection of HPA antibodies by anti-GPIa, anti-GPIba and anti-GPIIb/IIIa coupled microbeads. Anti-HPA-1a, anti-HPA-3a, and anti-HPA-5b are standard serum from NIBSC, anti-HPA-1b, anti-HPA-2b and anti-HPA-5a sera are clinic samples confirmed by MAIPA method. MFI values over cut-off value were considered as positive for the antibody

The HPA genotypes of three voluntary blood donors (P2, P3 and P4) using for specificity and sensitivity analysis were P2: HPA-1aa, HPA-2aa, HPA-3ab, HPA-4aa, HPA-5aa, HPA-15aa; P3: HPA-1aa, HPA-2ab, HPA-3aa, HPA-4aa, HPA-5aa, HPA-15bb; P4: HPA-1aa, HPA-2aa, HPA-3bb, HPA-4aa, HPA-5aa, HPA-15aa. The specificities of coupled beads detecting HPA antibodies were validated by anti-HPA-1a, anti-HPA-3a, and anti-HPA-5b standard sera, and serum samples we previously collected containing HPA-1b, HPA-2b, HPA-5a, anti-HPA-15a, or anti-HPA-15b antibodies, respectively (Fig. 2). All antibodies were validated except beads coated by anti-CD109. The MFI values of anti-HPA-1a, anti-HPA-2b, anti-HPA-3a, anti-HPA-5a serum samples reacted with the corresponding coupling beads were significantly higher than the cut-off value, indicating the Luminex assay can specifically identify HPA-1a, HPA-2b, HPA-3a and HPA-5a antibodies. In addition, because the three platelet donors were both HPA-5aa and HPA-1aa, HPA-5b standard positive serum, the HPA-1a standard positive serum could not react with the corresponding coupled beads, and the MFI was lower than the negative control, implying that the detection is not interfered by other HPA system antibodies. All serum sample results were replicated with the MAIPA assay and results were consistent, indicating that detection of HPA antibodies by Luminex assay is feasible. Anti HPA-15 showed negative results (data not show) when reacted with each platelet, which was replicated after coupling different CD109 antibodies to microbeads, which implied that using the microbeads to detect anti-HPA 15 was unsuccessful.

**Fig. 3:**
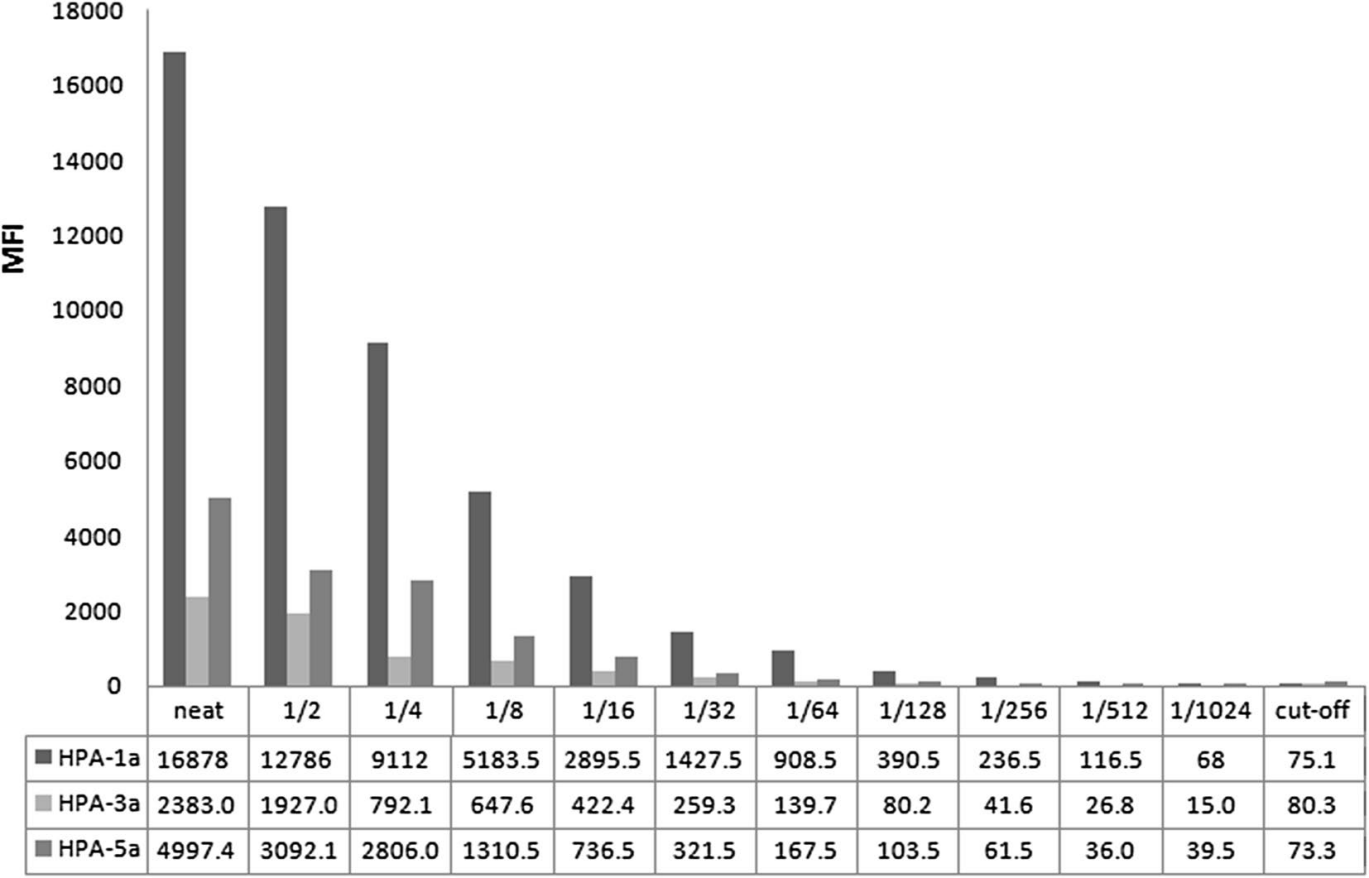
MFI values of serial dilutions of anti-HPA-1a, anti-HPA-3a standard sera, and anti-HPA-5a positive serum detected by the Luminex assay. The values in the table were the MFI measured by the Luminex beads

The sensitivity assay showed that the MFI value reached to 16,878.5 for HPA-1a antibody detection by the microbeads coupled with anti-GPIIb/IIIa (Fig. 3). The MFI value gradually decreased with the series of dilution sera and the MFI values of 1:2, 1:4, 1:8, 1:16, 1:32, 1:64, 1:128, and 1:256 diluted anti-HPA-1a standard sera were 12,786.5, 9112, 5183.5, 2895.5, 1427.5, 908.5, 390.5, and 236.5, which were significantly higher than the cut-off value (75.1). The sensitivity of anti-HPA-1a detection was 1:512 (0.195 IU/mL) using Luminex assay and was 1:64 (1.56 IU/mL) using MAIPA method as previously reported [13]. The sensitivity of anti-HPA-3a and anti-HPA-5a detection was 1:64 and 1:128, respectively, using the Luminex beads assay.

### Specificity of Luminex assay detecting HLA antibody

The W6/32 coupled beads were used to detect the HLA antibody and compared with the Luminex-PRA method and MAIPA method. Of the 163 samples, the Luminex method showed 44 samples positive for HLA-I antibody. There were no discrepancies among the results obtained from Luminex-PRA method and MAIPA method (data not show).

### Detecting HPA and HLA antibodies from clinical samples simultaneously

All anti-GP coupled beads except anti-CD109 were mixed to detect HPA and HLA antibodies from 44 serum samples, and all the samples were also tested by MAIPA. From the HPA genotyping of apheresis samples, all 8 negative controls showed negative results for anti-GPIa, anti-GPIba, anti-GPIIb/IIIa and anti-HLA coupled beads (Table 1). The cut-off values were listed in Table 2.

**Table 1:** HPA genotypes of the individuals used in the HPA antibodies detection

	HPA-1	HPA-2	HPA-3	HPA-4	HPA-5	HPA-15
G1	aa	ab	ab	aa	aa	ab
G2	aa	ab	aa	aa	aa	bb
G3	aa	aa	bb	aa	aa	aa
G4	aa	aa	ab	aa	ab	bb

G1–G4 are platelet donors

**Table 2:** Results of clinic samples determined by the Luminex assay

Sample	Platelet	MFI				Luminex results	MAIPA results
		GPIa/IIa (20)	GPIba/IX (36)	HLA (50)	GPIIb/IIIa (66)		
S2	G1	83.59	414.37	1399	72.18	Anti-HLA + anti-HPA-2b	Anti-HLA + anti-HPA-2b
	G2	89.70	468.21	438.5	108.04		
	G3	52.42	71.95	3341	86.68		
	G4	46.49	69.51	1442	58.83		
S3	G1	71.05	36.19	1390	70.60	Anti-HLA + anti-HPA-5b	Anti-HLA + anti-HPA-5b
	G2	57.32	68.15	2469	74.08		
	G3	83.57	43.42	2776.5	111.12		
	G4	941.90	71.25	1395	71.52		
T5	G1	56.06	21.63	45.19	3430.30	Anti-HPA-1a	Anti-HPA-1a
	G2	59.48	43.65	116.05	1053.89		
	G3	69.32	53.31	71.13	1318.20		
	G4	33.28	36.64	45.39	3997.51		
S7	G1	1402.07	39.17	109.89	1355.66	Anti-HPA-5a + anti-HPA-1a	Anti-HPA-5a + anti-HPA-1a
	G2	1410.06	34.86	111.47	1406.88		
	G3	1114.12	40.45	33.44	1297.48		
	G4	1498.50	79.59	106.75	1810.53		
T8	G1	33.77	41.91	103.64	1347.68	Anti-HLA + anti-HPA-3a	Anti-HLA + anti-HPA-3a
	G2	52.63	90.44	3675.49	1764.84		
	G3	52.21	47.67	2065.35	92.82		
	G4	51.73	37.52	1078.52	1153.10		
S9	G1	76.06	79.44	559.08	876.67	Anti-HLA + anti-HPA-3b	Anti-HLA + anti-HPA-3b
	G2	76.41	69.50	5511.44	107.48		
	G3	61.12	79.00	1961.17	785.68		
	G4	69.75	83.96	3080.16	976.73		
T10	G1	69.80	23.18	71.49	77.42	Anti-HPA-5b	Anti-HPA-5b
	G2	74.64	38.32	69.92	44.98		
	G3	70.63	31.47	69.48	97.10		
	G4	1979.27	80.06	78.05	54.36		
S11	G1	50.80	47.89	28.64	423.74	Anti-HPA-1a	Anti-HPA-1a
	G2	56.66	71.67	37.95	1022.23		
	G3	93.93	80.44	64.35	1248.52		
	G4	43.16	35.45	72.41	1203.06		
S12	G1	35.40	22.88	3135.34	91.55	Anti-HLA	Anti-HLA
	G2	41.92	74.05	14,619.87	130.06		
	G3	31.82	68.35	11,924.19	85.82		
	G4	54.81	29.88	9249.19	83.82		
Cut-off	132.29	85.19	131.97	163.46	132.29		

The table showed the results of 9 positive clinic samples determined by the Luminex assay. The Platelet represents the platelet donors (G1–G4) reacting with the samples. GPIa/IIa (20), GPIba/IX (36), HLA (50), and GPIIb/IIIa (66) are the monoclonal antibody coupled microbeads, the number in the brackets are the beads code. MFI represents the MFI values of coupled-beads reacted with the responding serum sample-platelet complex measured using a Luminex 100 instrument. Luminex results was inferred from the MFI value and platelet donor genotype; MAIPA was applied previously results. The cut-off MFI value is defined as the mean value of the negative control + 3SD value, and ≥ cut-off value is considered as positive

Of the 44 detected samples, 9 samples showed apparently higher MFI values than cut-off value, indicating that the samples were positive for HLA or HPA antibodies; but the other 35 samples showed negative results when reacted with the microbeads mixture. Of the nine positive samples, eight were from ISBT platelet workshop (S2, S3, T5, S7, T8, S9, T10, and S11), and one (S12) was from our lab. Four samples (S2, S3, S9, and T8) were observed positive for both HPA and HLA antibodies (Table 2), indicating that antibodies against HPA and HLA can be simultaneously detected in a single well. The samples containing HLA or HPA antibodies did not affect the specificity. One sample (S7) showed positive for both anti-HPA-1a and anti-HPA-5a, indicating lack of cross-reactivity in the presence of multi-HPA antibodies. These results were replicated with the MAIPA assay (Table 2).

## Discussion

The HPA systems are highly polymorphic in both healthy and disease states. For example, HPA-1a and HPA-5b antibodies are the most common antibodies causing immune thrombocytopenia in Caucasians, followed by HPA-1b, HPA-3b, HPA-15 system antibodies. However, HPA-3 and HPA-15 are the most common polymorphism systems in the Chinese Han population, while HPA-1a, and HPA-1b are not often present in HPA disorders in China. While many methods have been previously described, a more sensitive method to detect HPA antibodies based on the Luminex microbeads has been used to detect HPA antibodies in Caucasians and Japanese [9, 10, 12]. In this study, Luminex beads coupled with anti-GP and anti-HLA monoclonal antibodies were utilized to detect HPA-1, HPA-2, HPA-3, HPA-5, HPA-15 and HLA antibodies. In the specificity assay, the microbeads coupled with monoclonal antibody have been successfully used to detect HPA-1a, HPA-2b, HPA-3a, HPA-3b, HPA-5a, and HLA antibodies. Serum samples containing anti-HPA-1b oranti-HPA-5b have not been identified because no HPA-1b or HPA-5b-homozygote platelet donors used in the specificity assay, which imply that the anti-HPA-1b and anti-HPA-5b antibodies cannot affect the detection of anti-HPA-1a and anti-HPA-5a. However, this method failed to detect HPA-15 antibody. Chong et al. [9] have reported that HPA-15 antibody is difficult to detect in vitro because the expression of CD109 glycoprotein is very low and easy to degrade after storage, so it is extremely difficult to detect HPA-15 in vitro.

The sensitivity of the Luminex bead technology is significantly higher than that of MAIPA assay by detecting serial dilutions of anti-HPA-1a standard serum, which may have important significance in HPA-1a antibody detection, especially with low concentration. In addition, only ≤ 1:64 dilution for anti-HPA-3a could be detected, this may be due to the low affinity of anti-HPA-3a binding to corresponding glycoproteins monoclonal antibody, which was similar to the previous study reported by Fujiwara et al. [12]. We also compared the results using the Luminex assay and MAIPA assay for clinical samples and ISBT platelet workshop samples. There was no discrepancy between the results of the two methods, and multiple HPA or HLA antibodies in one sample can be identified successfully, which indicated that the beads mixture is suitable for detection of HPA and HLA antibodies with the microbeads-based assays.

Compared with MAIPA assay, the Luminex assay in this study could improve the sensitivity for the detection of the HPA antibodies and is also rapid with results available in about 3 h. In addition, different kinds of antibodies could be detected clearly by the coupled beads mixed together, and no cross-reactivity was observed containing HLA and multi-type of platelet antibodies, which, undoubtedly, could save the sample volume and make the assay more efficient. Due to the absent of HPA-1b platelet, one anti-HPA-1b positive serum we obtained before could not be confirmed in the Luminex assay. Although HPA-4, HPA-6, and HPA-21 have also been reported have polymorphisms in the Chinese population, but the polymorphisms are extremely low, and the corresponding HPA antibody positive sera are rare, which have not been reported yet in our lab, so we did not detect these antibodies. Therefore, it will be necessary to further evaluate this assay with more platelet samples or recombinant HPA antigen to validate the bead assay for rare HPA antibody detection.

## Conclusions

The HPA and HLA systems are complex and have clinical significance in many diagnosis and management of these disorders. However, there are still no corresponding effectively method to simultaneously identify antibodies against HPA and HLA. The results of our study show that the Luminex beads coupled with anti-GP and HLA antibodies could be successfully used to detect HPA and HLA antibodies simultaneously, especially with high sensitivity in detecting HPA antibody. The Luminex assay may be helpful for the diagnosis of HPA and HLA antibodies, and it will be interesting to assess the wider utility of this assay for the detection of other alloantibodies in the laboratories.

## Data Availability

Not applicable.

## Abbreviations

HLA: human leukocyte antigen
HPA: human platelet antigen
GP: platelet membrane glycoproteins
MAIPA: monoclonal antibody immobilization of platelet antigens assay
NAIT: neonatal alloimmune thrombocytopenia
PTP: post-transfusion purpura
PTR: platelet transfusion refractoriness
SPRCA: solid phase red cell adherence
ICFA: immuno-complex capture fluorescence analysis
GASA: gel antigen-specific assay
EQA: external quality assessment
